# Human Intrinsic Factor Expression for Bioavailable Vitamin B_12_ Enrichment in Microalgae

**DOI:** 10.3390/biology7010019

**Published:** 2018-02-19

**Authors:** Serena Lima, Conner L. Webb, Evelyne Deery, Colin Robinson, Julie A. Z. Zedler

**Affiliations:** 1 Industrial Biotechnology Centre, School of Biosciences, University of Kent, Giles Lane, Canterbury, Kent CT2 7 NK, UK; serena.lima@unipa.it (S.L.); C.l.webb@kent.ac.uk (C.L.W.); E.Deery@kent.ac.uk (E.D.); C.Robinson-504@kent.ac.uk (C.R.); 2 Dipartimento dell’Innovazione Industriale e Digitale, Università degli Studi di Palermo, Viale delle Scienze Ed. 6, 90128 Palermo, Italy; 3 Copenhagen Plant Science Centre, Department of Plant and Environmental Sciences, University of Copenhagen, Thorvaldsensvej 40, 1871 Frederiksberg C, Denmark

**Keywords:** *Chlamydomonas*, vitamin B_12_, cobalamin, intrinsic factor, microalgae, nuclear transformation, recombinant protein, dietary supplements, functional foods

## Abstract

Dietary supplements and functional foods are becoming increasingly popular complements to regular diets. A recurring ingredient is the essential cofactor vitamin B_12_ (B_12_). Microalgae are making their way into the dietary supplement and functional food market but do not produce B_12_, and their B_12_ content is very variable. In this study, the suitability of using the human B_12_-binding protein intrinsic factor (IF) to enrich bioavailable B_12_ using microalgae was tested. The IF protein was successfully expressed from the nuclear genome of the model microalga *Chlamydomonas reinhardtii* and the addition of an N-terminal ARS2 signal peptide resulted in efficient IF secretion to the medium. Co-abundance of B_12_ and the secreted IF suggests the algal produced IF protein is functional and B_12_-binding. Utilizing IF expression could be an efficient tool to generate B_12_-enriched microalgae in a controlled manner that is suitable for vegetarians and, potentially, more bioavailable for humans.

## 1. Introduction

There is an increasing awareness of the effect of diet on human health and, in particular, in the development of illness and chronic disease. This has led to an upward trend in consumer demand for naturally occurring bioactives that can be delivered in the form of dietary supplements or incorporated into functional foods. One of the common bioactives is vitamin B_12_.

Vitamin B_12_ or cobalamin (B_12_), is an essential cofactor produced by certain prokaryotes via two main complex biosynthetic pathways [1]. In humans, B_12_ is an essential cofactor required for a range of cellular metabolism functions (e.g., DNA synthesis and methylation, mitochondrial metabolism) [2]. B_12_ uptake and transport from ingestion to the blood stream is a multi-step complex pathway [3]. One of the proteins playing a key role in this process is the human protein intrinsic factor (IF). The glycoprotein binds free B_12_ in the ileum and the IF-B_12_ complex is then recognized by the cubam receptor complex facilitating receptor-mediated endocytosis [4]. The lack of a functional IF protein leads to the severe autoimmune disease pernicious anaemia [3]. Less severe but rather common cases of B_12_ deficiency are typically found in children, pregnant women and the elderly. Vegetarians and vegans are also at a higher risk for B_12_ deficiency, as higher plants do not produce or uptake B_12_ and are, therefore, not a dietary source of B_12_ [5]. To counteract the lack of dietary B_12_ intake, a wide range of supplements are commercially available. For instance, microalgal *Chlorella* supplements were found to contain amounts of B_12_ varying from trace amounts up to 415 μg per 100 g dry weight [6].

Microalgae are versatile, unicellular photosynthetic organisms that are attracting increasing attention. Despite edible microalgae having a long tradition as a food source, large scale cultivation has only become widespread since the middle of last century [7,8]. Potential exploitations of microalgae are as diverse as the polyphyletic group itself. In the context of this study, three main applications should be mentioned: (I) microalgae for functional foods (due to their high protein content, anti-oxidative properties and other potential health benefits) and as a vegetarian source of bioactive supplements [9,10,11]; (II) microalgae as a novel recombinant biotechnological host [12,13,14,15]; and (III) microalgae as a source of natural high-value compounds (recently reviewed in [12,16]).

These themes of microalgal applications are brought together in this proof-of-concept study by testing the possibility of enriching B_12_ in microalgae using the human IF protein. To this end, nuclear transformants of the green model alga *Chlamydomonas reinhardtii* expressing IF were generated. The IF protein was successfully expressed and the addition of a signal peptide from arsylsulfatase resulted in the efficient secretion of IF to the medium. The supplementation of the culture medium with B_12_ led to a higher IF abundance in the generated microalgal strains. This proof-of-concept shows microalgae are a viable host for the production of a vegetarian IF source for B_12_ enrichment. This could pave the way for developing a vegetarian source of bioavailable B_12_ for dietary supplements and the enrichment of functional foods.

## 2. Materials and Methods

### 2.1. Construct Design and Plasmid Construction

**Figure 1:**
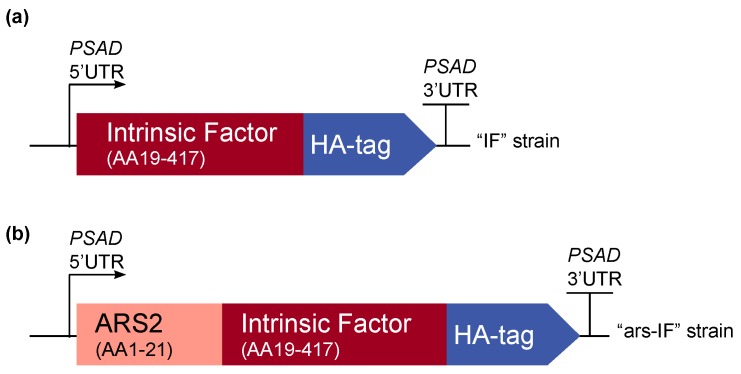
Overview of the intrinsic factor (IF) expression cassettes used for *C. reinhardtii* transformation. The strains expressing the constructs have been named “IF” and “ars-IF” respectively. The 3′ and 5′ untranslated regions of the *C. reinhardtii* PSAD gene are used for gene expression. (**a**) The N-terminal native signal peptide of the human IF protein was removed and the full length protein was codon-optimized for *C. reinhardtii* nuclear expression. A C-terminal human influenza hemagglutinin (HA) epitope tag was added to the protein for detection purposes. (**b**) Additionally, a second version of IF was constructed with the secretion signal peptide of arsylsulfatase ARS2.

The amino acid sequence of the human intrinsic factor (Uniprot KB: P27352) was codon-optimized for nuclear expression in the green alga *C. reinhardtii* using the software GeneDesigner 2.0 (Atum, Newark, CA, USA) [17]. The N-terminal native signal peptide (amino acid sequence (AA) 1–18) was removed and a C-terminal human influenza hemagglutinin (HA) epitope tag (AA sequence: YPYDVPDYA) was added for detection of the protein. Additionally, a second construct was made with an N-terminal secretion signal peptide of the *C. reinhardtii* native arsylsulfatase ARS2 (AA 1–21, Uniprot KB: Q9ATG5) [18]. The synthetic genes were custom synthesized by Genscript (Piscataway, NJ, USA) and cloned into the pCrEX1 nuclear expression vector (based on pSR108 [19]) using the restriction sites *SapI* (*LguI*) and *BglII*. Figure 1 gives an overview of the expression cassettes. The correct construct assembly has been verified by sequencing. All plasmids were amplified in *Escherichia coli* DH5α and purified for transformation of *C. reinhardtii*.

### 2.2. Strains and Cultivation Conditions

The *C. reinhardtii* strain CC-849 (cw10, mt−) was obtained from the Chlamydomonas Resource Center (University of Minnesota, Minneapolis, MN, USA). All strains were maintained at 25 °C and approximately 50 μE continuous illumination on 2% Tris-Acetate-Phosphate (TAP) medium [20] agar plates with a modified trace element recipe [21]. Liquid cultures were grown in TAP medium at 25 °C, 110 rpm shaking and approximately 100 μE continuous illumination. When TAP medium was supplemented with vitamin B_12_ (20–100 μg·L^−1^), commercially obtained cobalamin (Sigma-Aldrich, Dorset, United Kingdom) was added from a 0.1 g·L^−1^ stock solution. 

Initial screens for positive transformants were performed in 24 well plates. The wells were inoculated with single colonies and grown for 5 days before analysis. For protein expression analysis, 100 mL pre-cultures were inoculated from a plate. After 6 days, 100 mL cultures were re-inoculated to an OD_750_ of 0.01. Cells were subsequently harvested at mid to late log phase. For protein concentration and B_12_ assays, 1 L cultures were inoculated to an OD_750_ = 0.02 from a 1 week-old 100 mL pre-culture and grown to a mid-log phase stage before processing.

### 2.3. Nuclear Transformation

For nuclear transformation, 1 μg of the respective plasmid was linearized by cutting with *EcoRI*. A nuclear glass-bead transformation protocol was used based on a previously described method [22,23]. In brief, a 200 mL culture of CC-849 was grown to early log-phase and resuspended in 2 mL fresh TAP medium. Per transformation, 300 μL concentrated cells and 1 μg of linearized plasmid were used. The cell-DNA mixture was added to approximately 300 mg 400–600 μm acid-washed glass beads (Sigma-Aldrich, Dorset, United Kingdom) and agitated on a vortex for 15 s on maximum speed. The cells were diluted with TAP medium to a final volume of 10 mL and grown for approximately 18 h. After resuspension of the cell pellets in 500 μL TAP medium, soft agar (0.5%) was added and the mixture spread on TAP plates containing 10 μg·mL^−1^ Zeocin (InvivoGen, Toulouse, France) for selection of transformants that incorporated the construct containing the *ble* marker [19]. After approximately 8 days, colonies were re-streaked and subsequently analyzed for the presence of the gene of interest.

### 2.4. PCR Analysis

Transformant colonies were screened for the presence of the gene of interest using polymerase chain reaction (PCR). A gene fragment was amplified from genomic DNA using a standard protocol with Phire Plant Direct Master Mix (Thermo Fisher Scientific, Loughborough, United Kingdom). The following primers were used to amplify a 600 base pair fragment confirming the presence of the IF/ars-IF expression cassette: IF-fragF (5′-3′) CAGCATGAAGATTAAGGACA and IF-fragR (5′-3′) GTAGTACTGCGTGAAGTTG.

### 2.5. Preparation of Cellular Lysates and Medium Samples for Protein Expression Analysis

For protein expression analysis, cultures were grown as indicated in Section 2.2. Cellular lysates were prepared by resuspending an equivalent of OD_750_ = 1 cells in mid to late log phase in 100 μL 10 mM Tris-HCl (pH 8.0). To analyze the medium, 1 mL of culture was spun down at 20,000× *g* for 5 min and the cleared supernatant transferred to a separate tube.

### 2.6. Protein Concentration and Ion Exchange Chromatography

1 L mid-log phase cultures were harvested at 4000× *g* for 20 min at 4 °C. For protein concentration from the medium, the culture supernatant was filtered through Whatman filter paper to remove any debris and subsequently through a 0.2 μm membrane to remove any residual cells. The pre-purified medium fraction was subsequently loaded on the column.

For concentration of cellular proteins, the harvested cells were resuspended in lysis buffer (20 mM Tris-HCl (pH 7.2), 5% glycerol, 20 μg·mL^−1^ DNaseI (Roche, Mannheim, Germany), EDTA-free protease inhibitor (Roche)) and sonicated (3 cycles of 30 s sonication and 30 s on ice). The lysate was cleared by ultracentrifugation (Beckmann TLA-100.3 rotor (Beckman, Lismeehan, Ireland), 70,000 rpm, 30 min) and the soluble supernatant loaded on the column.

For ion exchange chromatography, 10 mL columns (Q Sepharose Fast Flow, GE Healthcare, Little Chalfont, United Kingdom) were freshly prepared for each purification. A vacuum pump was used to pass the medium samples through the column. After loading of the sample (medium or cellular lysate), the column was washed with a Tris based buffer (20 mM Tris-HCl (pH 7.2), 5% glycerol, 1 mM MgCl_2_, 5 mM dithiothreitol) containing 50 mM NaCl. Protein fractions were then eluted with buffer containing rising concentrations of NaCl (100 mM, 150 mM, 200 mM, 300 mM, 400 mM and 1 M NaCl).

### 2.7. Vitamin B_12_ Assay

The bioassay for the detection of B_12_ content in the protein fractions from ion exchange chromatography is based on the *Salmonella enterica* serovar Typhimurium LT2 strain AR2680 (*metE^–^*, *cbiB*^–^). Details of the assay are described elsewhere [24]. In very brief, this *Salmonella* strain cannot synthesize B_12_ de novo (CbiB^–^) and has no B_12_-independent methionine synthase (MetE^–^). Thus, growth of this strain is dependent on an external source of vitamin B_12_ as a co-factor for the vitamin B_12_-dependent methionine synthase MetH. The plaque size (growth) is dependent on the amount of externally supplemented vitamin B_12_, and can thus be used to semi-quantify Vitamin B_12_ levels in the sample. A calibration curve using 10 μL of 0.001 μM, 0.01 μM, 0.1 μM and 1 μM B_12_ solutions led to the equation used to calculate the B_12_ content in the analyzed samples:

vitamin B_12_ concentration (μM) = 0.0029 × (plaque diameter in cm)^5.4616^ (1)

Unpaired Student’s *t*-tests were performed using the software Prism (version 7.0, GraphPad Software, La Jolla, CA, USA). Values were considered statistically significant for *p* < 0.05.

### 2.8. Reducing SDS-PAGE, Native PAGE and Western Blotting

Samples were separated by electrophoresis on 12% polyacrylamide gels using a Bio-Rad Mini Protean Gel system (Bio-Rad Laboratories, Watford, United Kingdom). For reducing, denaturing sodium dodecyl sulfate polyacrylamide gel electrophoresis (SDS-PAGE), samples were boiled with Laemmli buffer containing β-mercaptoethanol at 95 °C for 5 min (crude cellular lysates and medium samples) or 10 min at 50 °C (fractions from ion exchange chromatography). For native analysis, samples were not boiled and no sodium dodecyl sulfate nor β-mercaptoethanol was used in samples, gels or running buffer. The polyacrylamide gels were subsequently immunoblotted using antibodies against the C-terminal HA-tag (Sigma-Aldrich, Dorset, United Kingdom) and against the C-terminus of the IF protein (abcam) to detect the protein of interest.

### 2.9. Densitometric Analysis

Densitometry analysis of immunoblots was performed using ImageLab Software Version 4.1 (Bio-Rad Laboratories, Watford, United Kingdom). The relative abundance of the IF protein was calculated in relation to the protein band found without supplementation of B_12_ to the medium. For the ars-IF strains, the calculated abundance was normalized to the OD_750_ of the cultures measured at harvesting to account for growth differences. This correction was not performed for the IF strains as equal amounts of cellular lysate standardized to OD_750_ were loaded on the gel.

## 3. Results

### 3.1. Generation of C. reinhardtii Nuclear Transformants “IF” and “ars-IF”

**Figure 2:**
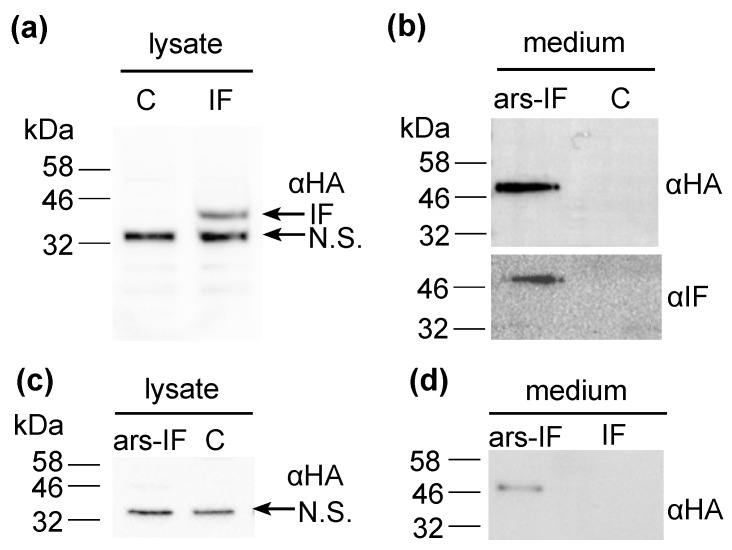
Expression of human intrinsic factor (IF) protein and ars-IF in *C. reinhardtii* CC-849 detected by immunoblot analysis using HA (αHA) and intrinsic factor (αIF) antibodies, respectively, in the culture medium and cellular lysates. (**a**) Cellular lysates of IF strain expressing the protein. The protein band is indicated with an arrow (IF). A second, non-specific band cross-reacting with the HA antiserum is indicated (N.S.). (**b**) Culture supernatant (medium) of transformed CC-849 strain expressing ars-IF. ‘C’ indicates a negative control (cellular lysate and supernatant of the untransformed CC-849 strain). (**c**) Cellular lysates of ars-IF strain and CC-849. (**d**) Culture medium of ars-IF and IF strain.

For this study, constructs for nuclear transformation of a wall-deficient *C. reinhardtii* strain (CC-849) were assembled with the human gene encoding the mature sequence of the B_12_-binding protein IF flanked by 5′ and 3′ untranslated regions (UTRs) of the PSAD gene. Two constructs were generated—one for cytoplasmic expression (Figure 1a) encoding IF and a second version with the addition of an N-terminal signal peptide from ARS2 (Figure 1b). This signal peptide has previously been shown to enable secretion to the medium in *C. reinhardtii* of recombinant proteins [18]. Additionally, a C-terminal HA epitope tag was added to both proteins for detection purposes. The *ble* marker downstream of the gene of interest on the pCrEX1 plasmid was used to select for positive transformants after transformation by agitation of a DNA-cell mixture with glass beads (modified from previously described methods [22,25], see Section 2.3). The presence of the IF cassette was verified by PCR (Figure S1) in colonies that grew on Zeocin after transformation. Positive candidates based on PCR results were then subjected to immunoblot analysis to screen for expression of the IF protein. Two strains with the respective constructs were found to express detectable levels of IF (Figure 2). The strains were named “IF” and “ars-IF” (Figure 1) and subjected to further analysis.

### 3.2. Expression of Human Intrinsic Factor in *C. reinhardtii*

The expected size of the mature size IF based on its amino acid sequence is 44.6 kDa. The IF protein expressed in the IF strain has a similar size migrating on an SDS-PAGE slightly faster than the 46 kDa molecular weight standard (Figure 2a). Additionally, a second non-specific band cross-reacting with the HA antiserum was detected in cellular lysates of both, the IF and the control strain around 32 kDa that we previously observed in *C. reinhardtii* cellular lysates [26]. The IF protein was expressed with an N-terminal ARS2 signal peptide in the ars-IF strain. The signal peptide and the mature IF protein have a combined predicted molecular weight of 46.3 kDa, however, the expected size of the mature protein after cleavage of the ARS2 signal peptide is the same molecular weight as the cytoplasmic IF protein with 44.6 kDa (this observation is discussed in more detail in Section 4.2). Immunoblot analysis of the ars-IF culture medium shows the protein migrating at a size around 50 kDa which is slightly bigger than expected. To further verify the band, which is not present in the CC-849 control strain (Figure 2b), the protein was also immunoblotted using an IF-specific antibody. With both antibodies, the same band was detected.

To determine the localization of the protein, culture supernatant (medium) and pelleted cells (cellular lysate) were immunoblotted separately. In the IF strain, the IF protein was only detected in cellular lysates (Figure 2a,d). In contrast to this, the IF protein expressed in the ars-IF strain with the ARS2 signal peptide was only found in the culture supernatant (Figure 2b), no protein was detected in cellular lysates (Figure 2c).

### 3.3. *C. reinhardtii*-Produced IF Stability Increases with External Vitamin B_12_ Supplementation

**Figure 3:**
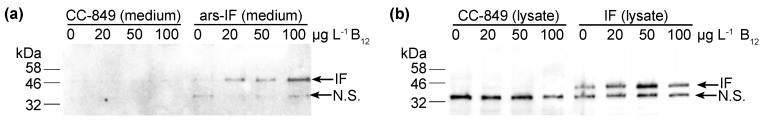
Accumulation of human intrinsic factor (IF) protein in *C. reinhardtii* ars-IF and IF cultures with increasing concentrations of vitamin B_12_ (cobalamin) supplemented TAP medium. (**a**) HA-immunoblot of medium harvested from CC-849 and ars-IF cultures grown to late log phase containing 0, 20, 50 and 100 μg·L^−1^ Vitamin B_12_. Equal amounts of culture supernatant (20 μL) loaded per lane. (**b**) HA-immunoblot of cellular lysates from CC-849 and IF cultures grown to late log phase with 0, 20, 50 and 100 μg·L^−1^ Vitamin B_12_ supplemented. Equal amounts of cellular lysate standardized by OD_750_ loaded. Mobility of IF protein indicated by arrow, N.S. denotes a non-specific protein cross-reacting with the HA-serum. Supplementary Figure S2 shows Coomassie stained SDS-polyacrylamide gels with total protein loaded for respective western blot samples.

To test the B_12_ binding capacities of the microalgal IF produced protein, the IF and ars-IF strains were grown in TAP medium supplemented with vitamin B_12_ at a concentration of 0, 20, 50 and 100 μg·L^−1^. As shown by immunoblotting, a correlation between higher B_12_ levels and the IF protein (ars-IF strain) in the medium is observed (Figure 3a). The protein abundance increases approximately 2.1 fold (±0.50) with the addition of 100 μg·L^−1^ B_12_ in the culture medium (Table 1). Similarly, based on densitometric analysis of relative protein abundance, the cytoplasmic IF protein in the IF strain is also estimated to be more abundant with B_12_ supplemented in the medium. However, here the highest abundance of IF is estimated to be at 50 μg·L^−1^ B_12_ (Figure 3b and Table 1).

### 3.4. Correlation of Vitamin B_12_ and IF Protein Levels

**Figure 4:**
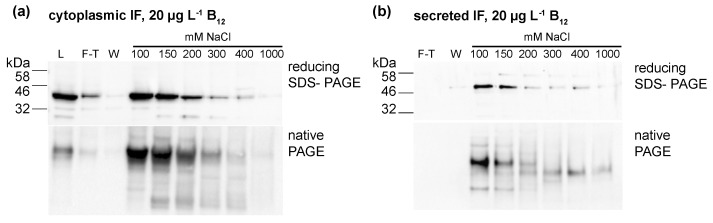
Protein fractions purified from (**a**) cellular lysates (human intrinsic factor (IF) strain) and (**b**) medium (ars-IF strain) HA-Immunoblots of reducing SDS- and native-PAGE show the presence of the IF protein in the separate fractions. L-lysate; F-T—flow-through; W-wash fractions (wash 1 and wash 2 as shown in Figure 4 were pooled); 100 mM–1000 mM: NaCl concentrations of eluted fractions.

To further investigate if the IF protein was binding B_12_, the protein was concentrated from cellular lysates (IF strain, CC-849 control) and the medium (ars-IF strain, CC-849 control). Ion exchange chromatography was used to concentrate and separate protein fractions from total cellular (IF strain) or secreted protein (ars-IF). Protein fractions were eluted with rising NaCl concentrations from 100 mM to 1 M NaCl. Both IF expressing strains and the negative control strain CC-849 were grown with either no B_12_ or 20 μg·L^−1^ B_12_ supplemented to the medium. The majority of the IF protein was eluted with 100–150 mM NaCl in both cases, the medium and the cytoplasmic IF version, as shown by immunoblot analysis of the individual protein fractions (Figure 4). Immunoblots of IF concentrated from cellular lysates of the IF-strain showed one prominent band of the protein with both reducing SDS-PAGE and native PAGE (Figure 4a). In the medium version, bands migrating differently in native PAGE than with reducing SDS-PAGE were observed (Figure 4b). 

**Figure 5:**
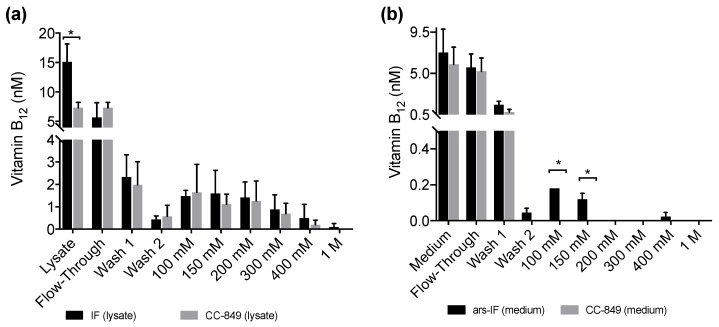
Vitamin B_12_ content of protein fractions purified from IF and ars-IF strains grown with 20 μg·L^−1^ supplemented TAP medium determined using a plaque assay of *Salmonella enterica* (*metE^–^ cbiB*^–^). Fractions were eluted with increasing concentrations of NaCl (100 mM to 1 M). Data show average values, error bars: standard error (*n* = 3). Statistically significant differences between the control and the respective strain for each fraction are indicated with an asterisk (Student’s *t*-test, *p* < 0.05). (**a**) B_12_ amounts detected in fractions of 1 L cellular lysates from IF and CC-849 strains. (**b**) B_12_ amounts detected in protein fractions purified from 1 L medium of ars-IF or CC-849 cultures.

All fractions, the lysates and medium samples were subsequently used in a B_12_ bioassay to quantify their B_12_ content. This bioassay is based on the growth dependency of a *Salmonella enterica* (*metE^–^ cbiB*^–^) strain on external B_12_ supplementation. All experiments were performed in triplicate and the B_12_ content found in the individual fractions is shown in Figure 5a for the IF-strain and Figure 5b for the ars-IF strain when cultures were supplemented with 20 μg·L^−1^ B_12_. In both cases, the highest B_12_ content was found in the lysate (medium), flow-through and wash fractions. Significant differences in B_12_ content between the control strain and the IF strain were only found in the cellular lysate (*p* = 0.01) however this observation was not supported by the eluted protein fractions (Figure 5a). In case of the ars-IF strain, significant differences were only found in the 100 and 150 mM NaCl eluted protein fractions (*p* = 0.02) (Figure 5b). All purifications from cultures grown without B_12_ supplemented in the medium did not contain B_12_ levels detectable with the bioassay. The elution pattern of the IF protein in these samples was, however, found to be overall the same as from cultures supplemented with B_12_. The majority of secreted IF protein was found to be cleaved or degraded without B_12_ supplementation as seen by SDS-PAGE analysis during the purification process (Figure S3b). Whereas a similar pattern, with and without B_12_ supplementation of the medium, was found for the cytoplasmic IF protein (Figure S3a).

## 4. Discussion

### 4.1. Expression and Efficient Secretion of a Human Glycoprotein Intrinsic Factor in Microalgae

In this study we have successfully expressed the human B_12_ receptor protein IF in *C. reinhardtii*. The protein was expressed from the nuclear genome as this allowed for secretion of the protein to the medium and potential glycosylation of the protein. Two different strains were generated for this study—one strain expressing the 44.6 kDa IF protein in the cytoplasm (IF-strain) and a second strain that secretes the protein to the medium (ars-IF). The ars-IF expressed IF protein has been found to be efficiently secreted to the medium by means of an ARS2 signal peptide that has previously been used to secrete a recombinant enzyme [18]. No protein was detected in the cellular lysate in the ars-IF strain (Figure 2c). This suggests more efficient secretion of the ars-IF than what has been reported for secretion of a luciferase using the same ARS2 signal peptide [18]. To date, only few studies on the secretion of recombinant proteins in microalgae are available. Most reports are from the green alga *C. reinhardtii* [18,27,28,29,30] or the diatom *Phaeodactylum tricornutum* [31,32].

### 4.2. Posttranslational-Modification of the Secreted IF Protein

Based on migration on SDS-PAGE gels, the secreted microalgal IF protein showed a consistently larger molecular size compared to the cytoplasmic protein (Figure 3, Figure 4 and Figure 5b,c). There are several reasons that could explain this. One possibility is the failure to cleave the signal peptide. However, the ARS2 signal peptide only adds approximately 1.7 kDa to the mature protein which seems unlikely to cause the observed shift of around 5 to 10 kDa. A previous study has used an ARS1 signal peptide for secretion of a protein to the medium and observed a similar pattern. In this study, the signal peptide was not found with mass fingerprint analysis suggesting that the non-cleavage of the signal peptide was unlikely [18].

Another possibility that could explain the size difference is the post-translational modification of the IF protein. This is likely as the human IF protein contains an *N-*glycosylation site [33] and is known to be highly glycosylated. When previously expressed in higher plants (*Arabidopsis thaliana*) glycosylation was observed and the protein detected had a similar size of 50 kDa to the algal expressed IF [34]. The glycosylation of IF seems to be non-essential for B_12_ and receptor binding [35], however, it may stabilize and protect the protein from degradation in the low pH and proteolytic conditions in the intestine. Not much is known about *N-*glycosylation in *Chlamydomonas* [27,36,37] and efforts to characterize the secreted IF protein in more detail were prevented by low expression levels. Treatment of the protein with a commercial deglycosylation enzyme cocktail (Protein Deglycosylation Mix II, New England Biolabs, Hitchin, United Kingdom) was inconclusive and we did not obtain sufficient amounts of pure protein for mass spectrometry (data not shown) from 1 L cultures (OD_750nm_ between 0.6 and 0.8). Low expression levels of recombinant proteins are to date a common problem seen in *Chlamydomonas* (previous studies have reported 0.25% of total cellular protein [38] or 0.25% of total soluble protein [30] for example). This is likely due to various factors such as random insertion of the construct in the nuclear genome and efficient silencing mechanisms [39]. Recent efforts to establish targeted DNA approaches (see [14,40] for recent reviews) such as CRISPR/Cas9 technology [41,42,43] for nuclear transformation in *C. reinhardtii* could be promising tools to increase recombinant protein yields allowing for a more detailed characterization of the algae-produced IF protein.

### 4.3. Vitamin B_12_ Enrichment in IF Expressing *C. reinhardtii* Strains

Eukaryotic microalgae do not produce B_12_ and more than half of microalgal species are thought to be B_12_ auxotrophs relying on B_12_ obtained from symbiotic relationships with bacteria [44,45]. The model alga *C. reinhardtii* used in this study has been shown to be B_12_ independent as it contains *METE* (B_12_-independent methionine synthase gene). However, the alga also contains the B_12_-dependent B_12_ methionine synthase MetH which is preferably used in the presence of B_12_ [44]. 

No B_12_ was detected in any protein fractions concentrated from cultures grown without B_12_ supplementation (Table S1) which is expected as the algae were grown axenically. When supplementing the medium with B_12_, increased amounts of the IF protein were detected in both strains, IF and ars-IF (Figure 3 and Table 1). This indicates that the IF protein is stabilized due to B_12_ binding. IF is a known B_12_ binding protein and formation of the holo-IF form stabilizes the protein [33]. Binding of B_12_ to the cytoplasmic IF protein presumes the cellular uptake of B_12_ from the medium into the cell. B_12_ transport into the cell in *Chlamydomonas* is existent, evident by their ability to utilize B_12_ [44,46] but molecular mechanisms of B_12_ uptake in microalgae have hardly been described [47]. The semi-quantitative B_12_ assay shows a significant difference of the total B_12_ amount accumulated in the IF strain lysates compared to a negative control strain (Figure 5a). However, this observation was not supported by the individual elution fractions. If the increased amount of B_12_ found in the IF lysates was directly due to binding to the IF protein, one would expect to see a significant difference in the elutions containing the majority of the IF protein (100 mM, 150 mM NaCl, Figure 4). However, no significant differences of any of the elution fractions were found for the IF and CC-849 control strain. Consequently, it is possible that the expression of IF in the algae does lead to an enrichment of intracellular vitamin B_12_, but further work is needed to support this hypothesis. 

With the ars-IF strain, the IF protein was purified from the medium where the background from other co-purified proteins is significantly lower than in the cellular lysates. Here, the fractions that contain the highest amount of IF protein show a significantly higher B_12_ content when compared with the control strain CC-849 (Figure 5b). 

We also observed that, in the flow-through and the wash fractions (Figure 5b), no significant differences between the ars-IF and the control strain is seen. This is likely to be due to the unbound B_12_ washing off and the B_12_ detected in subsequent elution fractions most likely being bound to protein. However, the B_12_ bioassay used in this study does not allow distinguishing between protein-bound B_12_ and free B_12._ Therefore, we cannot conclude if the B_12_ was bound to the secreted IF protein.

In the case of the cytoplasmic IF protein, there is a statistically significant difference between B_12_ concentrations measured in cellular lysates expressing IF and the control strain (Figure 5a). However, there was no significant difference observed in the elution fractions. One reason for this might be that the assay does not distinguish between B_12_ bound to the IF protein or other cellular proteins. Other enzymes already known to be associated with intracellular B_12_ metabolism in algae will most likely interact with internalized B_12_ [45]. This could explain the high background found in the protein elutions from both cellular lysates, CC-849 and IF strain (Figure 5a). Additionally, a relative decrease of cytoplasmic IF was observed from cultures supplemented with 50 μg·L^−1^ B_12_ to cultures supplemented with 100 μg·L^−1^ B_12_ (Figure 3b and Table 1). Although we can only hypothesize, this decrease could be due to concentration-dependent toxicity of the protein and therewith associated cellular regulatory processes. Additionally, due to the random insertion of the expression cassette in the nuclear genome, there is the possibility that other cellular functions may be affected.

### 4.4. Intrinsic Factor for Developing B_12_-Enriched Microalgae

In this study we expressed IF in the eukaryotic microalgae *Chlamydomonas* exploring the potential of enriching bioavailable B_12_ using a microalgal host. We propose that this model alga could be a good chassis for future production of microalgal functional foods and dietary B_12_ containing supplements. For example, recently it has been shown that cyanobacteria produce a different chemical variant of B_12_ [48]. This chemical variant of B_12_ (also known as pseudocobalamin) binds with a much lower affinity to B_12_ receptor proteins such as IF making it less bioavailable for humans [49]. Eukaryotic microalgae, on the other hand, utilize the same B_12_ variants as humans, therefore, making them a better source of B_12_ supplementation. 

As shown recently for *Chlorella* supplements [6], currently available eukaryotic microalgal supplements can contain varying amounts of B_12_. This is most likely due to different cultivation conditions altering the composition of the associated bacterial biofilms that are the original source of B_12_ found in the supplements. In this respect, the establishment of closed, standardized growth systems, as previously shown for *Chlamydomonas* [50,51], allows for much more control over contamination compared to open systems and hereby also of the B_12_ content in the final product. In addition, efforts to engineer microalgal communities (reviewed recently in [12,52]) with symbiotic relationships, as found between *Mesorhizobium loti* and *Lobomonas rostrata* [53,54], could allow for a more controlled interaction, and thus flux of B_12_, of the microalga and associated bacteria. Finally, looking at the current market, IF, of animal (porcine) origin, is commercially available in some B_12_ supplements. This proof-of-concept study shows that microalgae are a potential vegetarian-friendly alternative to IF-B_12_ co-formulation in functional foods and dietary supplements.

## 5. Conclusions

Here we have shown that the human glycoprotein Intrinsic Factor can be expressed in *Chlamydomonas reinhardtii* from the nuclear genome. The addition of an *ARS2* signal peptide leads to efficient secretion to the medium. Our data suggest that the secreted IF protein directly binds B_12_, however, it is less clear if the cytoplasmic IF binds the cofactor or has an indirect effect on B_12_ enrichment in the IF strain. Our data also show a correlation of higher protein levels with higher B_12_ concentrations suggesting a potential stabilizing effect on the protein. This study is a first proof-of-concept utilizing a human B_12_ binding protein as a tool for enriching B_12_ in microalgae and can contribute to the future development of microalgal functional foods.

## Figures and Tables

**Table 1:** Relative human intrinsic factor (IF) protein expression levels in IF and ars-IF strains for different B_12_ concentration supplemented to growth medium (*n* = 3 ± standard error).

Vitamin B_12_ in Medium (μg·L^−1^)	ars-IF Strain (Medium) ^1^	IF Strain (Cytoplasmic) ^2^
0	1.0	1.0
20	1.1 ± 0.01	1.4 ± 0.15
50	1.6 ± 0.17	2.3 ± 0.17
100	2.1 ± 0.50	1.4 ± 0.11

^1^ Densitometric relative quantity of IF was normalized to the OD_750_ of the respective cultures measured when samples were harvested. ^2^ Equal amounts of cells, normalized to OD_750_, for all cultures were loaded on gel for immunoblotting.

## Supplementary Materials

The following are available online at www.mdpi.com/2079-7737/7/1/19/s1, Figure S1: PCR analysis of *Chlamydomonas reinhardtii* strains IF and ars-IF. Figure S2: Coomassie-stained polyacrylamide gels of samples shown in Figure 3. Figure S3: IF purified from cultures grown without B_12_ supplementation. Table S1: Vitamin B_12_ assay plaque diameters measured in different fractions from ion exchange chromatography and calculated Vitamin B_12_ content of samples (nM).
