# Supplementary material for: Human Intrinsic Factor Expression for Bioavailable Vitamin B12 Enrichment in Microalgae
Source: Biology (Basel). 2018 Feb 19;7(1):19. doi: 10.3390/biology7010019 (PMC5872045; doi:10.3390/biology7010019)
Supplement: Supplementary file 1 [file biology-07-00019-s001.pdf]

Supplementary Material

Human Intrinsic Factor Expression for Bioavailable Vitamin B₁₂ Enrichment in Microalgae

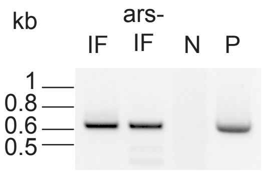

Figure S1. PCR analysis of *Chlamydomonas reinhardtii* strains IF and ars-IF. A 0.6 kilo base pair (kb) DNA-fragment of the intrinsic factor gene has been amplified from genomic DNA. Lanes show the transformant strains IF and ars-IF, CC-849 (N, negative control) and a positive control (P, plasmid DNA used for transformation mixed with CC-849 cells).

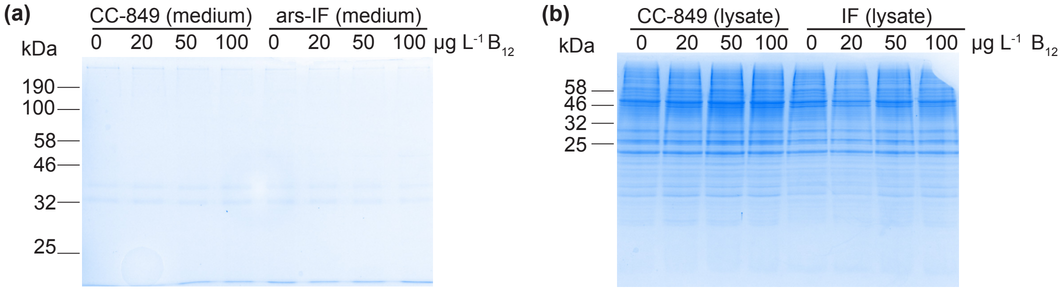

Figure S2. Coomassie-stained polyacrylamide gels of samples shown in figure 3. **(a)** medium harvested from CC-849 and ars-IF cultures grown to late log phase containing 0, 20, 50 and 100 µg L^{-1} Vitamin B_{12}. 20 µL of culture supernatant loaded per lane. **(b)** Cellular lysates from CC-849 and IF cultures grown to late log phase with 0, 20, 50 and 100 µg L^{-1} Vitamin B_{12} supplemented. Equal amounts of cellular lysate standardized by OD_{750} loaded.

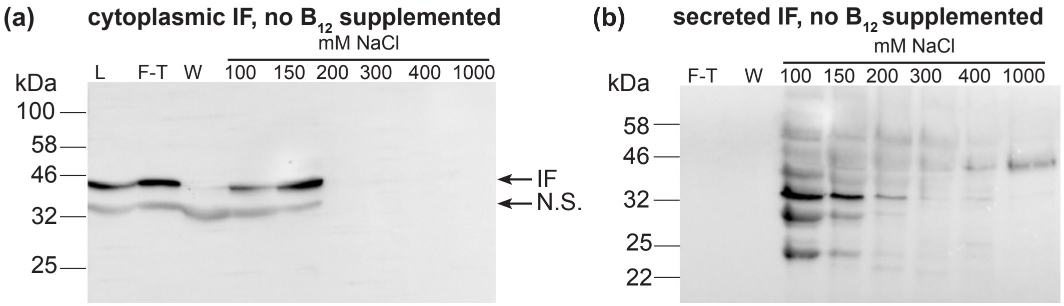

Figure S3. IF purified from cultures without B_{12} supplementation. **(a)** Cytoplasmic IF purified from IF cellular lysates. **(b)** Secreted IF purified from ars-IF cultures. Anti-HA immunoblots of fractions shown . L- lysate, F-T – flow-through; W-wash fractions (wash 1 and wash 2 as shown in figure 4 were pooled); 100 mM – 1000 mM: NaCl concentrations of eluted fractions. N.S. denotes non-specific protein band.

Table S1. Vitamin B_{12} assay plaque diameters measured in different fractions from ion exchange chromatography and calculated Vitamin B_{12} content of samples (nM).

ars-IF, medium (20 µg L^{-1} Vitamin B_{12} supplemented)

	plaque diameter (cm)			Vitamin B_{12} (nM)		
Fraction	replicate 1	replicate 2	replicate 3	replicate 1	replicate 2	replicate 3
Medium	0.95	1.25	1.25	2.19	9.81	9.81
Flow-Through	1.00	1.15	1.20	2.90	6.22	7.85
Wash 1	0.80	0.95	0.90	0.86	2.19	1.63
Wash 2	0.50	0.50	0.00	0.07	0.07	0.00
100 mM	0.60	0.60	0.60	0.18	0.18	0.18
150 mM	0.50	0.60	0.55	0.07	0.18	0.11
200 mM	0.00	0.00	0.00	0.00	0.00	0.00
300 mM	0.00	0.00	0.00	0.00	0.00	0.00
400 mM	0.50	0.00	0.00	0.07	0.00	0.00
1 M	0.00	0.00	0.00	0.00	0.00	0.00

ars-IF, medium (no Vitamin B_{12} supplemented)

	plaque diameter (cm)		
Fraction	replicate 1	replicate 2	replicate 3
Medium	0.00	0.00	0.00
Flow-Through	0.00	0.00	0.00
Wash 1	0.00	0.00	0.00
Wash 2	0.00	0.00	0.00
100 mM	0.00	0.00	0.00
150 mM	0.00	0.00	0.00
200 mM	0.00	0.00	0.00
300 mM	0.00	0.00	0.00
400 mM	0.00	0.00	0.00
1 M	0.00	0.00	0.00

CC-849, medium (20 µg L^{-1} Vitamin B_{12} supplemented)

	plaque diameter (cm)			Vitamin B_{12} (nM)		
Fraction	replicate 1	replicate 2	replicate 3	replicate 1	replicate 2	replicate 3
Medium	0.95	1.20	1.20	2.19	7.85	7.85
Flow-Through	1.00	1.20	1.10	2.90	7.85	4.88
Wash 1	0.60	0.80	0.85	0.18	0.86	1.19
Wash 2	0.00	0.00	0.00	0.00	0.00	0.00
100 mM	0.00	0.00	0.00	0.00	0.00	0.00
150 mM	0.00	0.00	0.00	0.00	0.00	0.00
200 mM	0.00	0.00	0.00	0.00	0.00	0.00
300 mM	0.00	0.00	0.00	0.00	0.00	0.00
400 mM	0.00	0.00	0.00	0.00	0.00	0.00
1 M	0.00	0.00	0.00	0.00	0.00	0.00

CC-849, medium (no Vitamin B_{12} supplemented)

	plaque diameter (cm)		
Fraction	replicate 1	replicate 2	replicate 3
Medium	0.00	0.00	0.00
Flow-Through	0.00	0.00	0.00
Wash 1	0.00	0.00	0.00
Wash 2	0.00	0.00	0.00
100 mM	0.00	0.00	0.00
150 mM	0.00	0.00	0.00
200 mM	0.00	0.00	0.00
300 mM	0.00	0.00	0.00
400 mM	0.00	0.00	0.00
1 M	0.00	0.00	0.00

IF, lysate (20 µg L^{-1} Vitamin B_{12} supplemented)

	plaque diameter (cm)			Vitamin B_{12} (nM)		
Fraction	replicate 1	replicate 2	replicate 3	replicate 1	replicate 2	replicate 3
Lysate	1.40	1.35	1.30	18.22	14.94	12.15
Flow-Through	1.00	1.20	1.15	2.90	7.85	6.22
Wash 1	0.85	1.00	1.00	1.19	2.90	2.90
Wash 2	0.65	0.70	0.75	0.28	0.41	0.60
100 mM	0.85	0.90	0.90	1.19	1.63	1.63
150 mM	0.95	0.95	0.70	2.19	2.19	0.41
200 mM	0.85	0.95	0.80	1.19	2.19	0.86
300 mM	0.70	0.90	0.75	0.41	1.63	0.60
400 mM	0.65	0.85	0.00	0.28	1.19	0.00
1 M	0.00	0.65	0.00	0.00	0.28	0.00

IF, lysate (no Vitamin B_{12} supplemented)

	plaque diameter (cm)		
Fraction	replicate 1	replicate 2	replicate 3
Lysate	0.00	0.00	0.00
Flow-Through	0.00	0.00	0.00
Wash 1	0.00	0.00	0.00
Wash 2	0.00	0.00	0.00
100 mM	0.00	0.00	0.00
150 mM	0.00	0.00	0.00
200 mM	0.00	0.00	0.00
300 mM	0.00	0.00	0.00
400 mM	0.00	0.00	0.00
1 M	0.00	0.00	0.00

CC-849, lysate (20 µg L^{-1} Vitamin B_{12})

	plaque diameter (cm)			Vitamin B_{12} (nM)		
Fraction	replicate 1	replicate 2	replicate 3	replicate 1	replicate 2	replicate 3
Lysate	1.15	1.20	1.20	6.22	7.85	7.85
Flow-Through	1.15	1.20	1.20	6.22	7.85	7.85
Wash 1 (15 mL)	0.80	1.00	0.95	0.86	2.90	2.19
Wash 2 (5 mL)	0.00	0.80	0.80	0.00	0.86	0.86
100 mM	1.00	0.70	0.90	2.90	0.41	1.63
150 mM	0.80	0.80	0.90	0.86	0.86	1.63
200 mM	0.70	0.85	0.95	0.41	1.19	2.19
300 mM	0.65	0.85	0.75	0.28	1.19	0.60
400 mM	0.00	0.70	0.60	0.00	0.41	0.18
1 M	0.00	0.00	0.00	0.00	0.00	0.00

CC-849, lysate (no Vitamin B_{12} supplemented)

	plaque diameter (cm)		
Fraction	replicate 1	replicate 2	replicate 3
Lysate	0.00	0.00	0.00
Flow-Through	0.00	0.00	0.00
Wash 1	0.00	0.00	0.00
Wash 2	0.00	0.00	0.00
100 mM	0.00	0.00	0.00
150 mM	0.00	0.00	0.00
200 mM	0.00	0.00	0.00
300 mM	0.00	0.00	0.00
400 mM	0.00	0.00	0.00
1 M	0.00	0.00	0.00
